# The Correlation between Early Stages of Life Exposed to Chinese Famine and Cognitive Decline in Adulthood: Nutrition of Adulthood Plays an Important Role in the Link?

**DOI:** 10.3389/fnagi.2017.00444

**Published:** 2018-01-10

**Authors:** Hongguo Rong, Yuandi Xi, Yu An, Lingwei Tao, Xiaona Zhang, Huiyan Yu, Ying Wang, Zhongsheng Qin, Rong Xiao

**Affiliations:** ^1^School of Public Health, Capital Medical University, Beijing, China; ^2^Jincheng People’s Hospital, Jincheng, China

**Keywords:** early life, Chinese Famine, adulthood, cognitive decline, nutrition, nutrient consumption pattern

## Abstract

**Objective:** The aim of this study was to investigate whether people exposed to the Chinese Famine in fetal period or in multiple stages of childhood are associated with cognitive decline in adulthood. Furthermore, the nutritional environment of adulthood was explored as an important factor in this correlation.

**Methods:** 1162 adults born between 1952 and 1964 were recruited. They were divided into five groups which were non-exposed group, fetal-exposed group, early childhood-exposed group, mid childhood-exposed group and late childhood-exposed group. Cognitive function was measured by using a comprehensive neuropsychological battery test, including Montreal cognitive assessment-Beijing version, mini-mental state examination, auditory verbal learning test, digit span forward, digit span backward, trail making test, and digit symbol test. Semi-quantified food frequency questionnaire (FFQ) was used to assess the dietary nutrition in their adulthood. The dietary nutrient consumption pattern was identified by Two-step and *K*-means cluster analysis.

**Results:** The significant differences in cognitive function were manifested in different groups. Compared with non-exposed group, subjects in fetal-exposed group had a higher risk of mild cognitive impairment (MCI) (OR 1.51 95% CI 1.02–2.23, *P* = 0.039) and global cognitive decline (OR 1.68 59% CI 1.02–2.77, *P* = 0.044). The similar result was also observed in subjects of early childhood-exposed group. Otherwise, subjects who were classified in high nutrient consumption pattern had higher risk of cognitive decline. Moreover, the higher consumption of several nutrients such as fat, carbohydrate and manganese were associated with worse performance on digit span forward, digit span backward, trail making test A, trail making test B and digit symbol.

**Conclusion:** Early stages of life exposed to the Chinese Famine were associated with higher risk of cognitive decline in adulthood. The stronger associations were manifested in the people with high nutrient consumption pattern. The consumption of fat, carbohydrate and manganese were associated with multiple domains cognitive decline.

## Introduction

Rapid demographic aging is becoming a vigilant public health issue in the developing countries including China (Kunna et al., 2017). Characterized by deficits in attention, executive function, working memory and information processing (Sampath et al., 2017), Mild Cognitive Impairment (MCI) is considered to be a critical stage intermediate period between normal aging and dementia. Data show that 20–30% of patients who were diagnosed as MCI will develop into dementia like Alzheimer’s disease (AD) in 2 years (Alzheimer’s Association, 2016; Nesteruk et al., 2016; Pena-Altamira et al., 2017). What are the earliest origins of AD? Based on epidemiological surveys and animal experiments over the past decades, studies support the idea that AD-related brain changes may originate from conception and early life (Albert, 2015; Seifan et al., 2015; Mormino et al., 2016; Rannikko et al., 2016). The fetal origin hypothesis indicates that adverse prenatal and postnatal environmental conditions increase the risk of future neurodegenerative disorders. Particularly, nutrition is one of the most important factors in early life. Malnutrition during fetal and early postnatal life may result in a permanent deficit of brain structure development. It prompts that malnutrition in early life may not only have the irreversible impact on catch-up growth in childhood, but also have the long-term effects on cognitive function in adulthood (Lucas, 2010).

Population study provides an opportunity to examine the relationship between early life nutrition and various adult diseases such as chronic disease and neurodegenerative disease. Because it is very difficult to observe the long-term consequences for decades in birth cohort, the study of people exposed to Famine in early life provides the indirect evidence of its long-term negative effect on people’s adult cognitive function. Studies of early gestation exposed to Dutch famine indicated that poor prenatal nutrition had the negative influences on the cognitive ability of people in their late adulthood (Rooij et al., 2010; Crookston et al., 2011). However, this association was not observed in the other Dutch famine families study, which suggested that there was no correlation between prenatal Famine exposure and cognitive decline at age of 59 among the male and female (de Groot et al., 2011). Therefore, whether early life nutrition is associated with cognitive decline in adulthood still needs to be further investigated. Meanwhile, additional studies are needed to fully elucidate what are the influencing factors in this correlation.

The most severe period of Chinese Famine generally accepted from 1959 to 1961. Compared to the other famines, Chinese Famine had the characteristics of long duration, wide range and severe degree. The age of people who exposed to Chinese Famine during fetal period or/and in early postnatal life are generally older than 55. With the increase in age-related cognitive impairment in China, it is urgent to find out effective ways of prevention. As is known, nutrition in adulthood is an important factor in chronic non-communicable disease. Therefore, adult diet, as a modifiable factor, might be a vital way of preventing cognitive decline in adulthood. The aim of this study was to investigate whether people exposed to the Chinese Famine in fetal period or in multiple stages of childhood are associated with cognitive decline in adulthood. Furthermore, the nutrition of adulthood was explored as an important factor in this correlation.

## Materials and Methods

### Study Population

Subjects in the present study come from an ongoing epidemiological survey program in our research group. This program is a multi-center survey study between 2013 and 2017. The exclusion and inclusion criteria of the study have been described in previous study (Wang C. et al., 2016). In this study, 1162 subjects came from Jincheng People’s Hospital in Shanxi province. This program was conducted according to the Declaration of Helsinki and ethically approved by the Ethics Committee of Capital Medical University (2013SY35). All subjects have signed informed consent at the beginning of the investigation.

### Famine Age Categories

Similar to previous study (Shi et al., 2017), according to the stage of life exposed to the Famine, participants in this study were categorized into five groups by their birthdates/dates of birth. Subjects in non-exposed group were born between October 1st, 1962 and September 30th, 1964 (*n* = 266, age = 53 ∼ 54). People in exposed groups were classified as follows: fetal-exposed group (born between October 1st, 1959 and September 30th, 1961, *n* = 210, age = 56–57), early childhood-exposed group (born between October 1st, 1956 and September 30th, 1958, *n* = 254, age = 59–60); mid childhood-exposed group (born between October 1st, 1954 and September 30th, 1956, *n* = 227, age = 61–62) and late childhood-exposed group (born between October 1st, 1952 and September 30th, 1954, *n* = 205, age = 63–64). Since the date of start and end of the Chinese Famine were not available, participants born between October 1st 1958 and September 30th 1959 and October 1st 1961 and September 30th 1962 were excluded in order to minimize the misclassification.

### Demographics

The structured questionnaire was used to collect the data of demographics, clinical characteristics and lifestyle risk factors of participants by face-to-face interview. The investigators were trained strictly before the formal investigation. The investigation of smoking status and alcohol consumption has set up three options which were never, former and current. Occupational categories classified as manual workers (e.g., manual laborers and farmers) and non-manual workers (e.g., technicians, managerial staff) based on one question with 9 occupational selection. History of chronic disease including hyperlipidemia, stroke, hypertension and coronary heart disease was collected by self-guided questionnaire and in accordance with clinical diagnosis by physicians. The formula of body mass index (BMI) is weight (kg)/height (m^2^).

### Blood Sample

Blood samples were collected during 8–10 am. Serum levels of total cholesterol (TC), triglyceride (TG), high-density lipoprotein cholesterol (HDL-C), and low-density lipoprotein cholesterol (LDL-C) were measured by standard enzymatic methods on TBA120-FR automatic biochemical analyzer (Toshiba Ltd., Japan).

### Dietary Assessment

Semi-quantitative food frequency questionnaire (FFQ) (He et al., 2009) including 33 food items (whole grain, legume and legume product, fruit and vegetables, chicken, red meat, cooking oil, tea, nuts, fish, eggs, milk and coffee, etc.) was used to estimate the dietary intake. The FFQ contained the information including quantity (weight or volume) and frequency (daily, weekly, monthly, yearly, or never) of foods consumption. The intake of nutrients were calculated by using the China Food Composition (2009) (Yang et al., 2009). Two-Step and *K*-means cluster analysis method were used to explore the dietary nutrient consumption patterns according to the similar research (Kim et al., 2015). Briefly, we analyzed subjects’ average daily intake of each nutrient according to the China Food Composition (2009). Compared with the reference nutrients intake in new Dietary guidelines for Chinese elderly residents (2016) (Wang S.S. et al., 2016) and Chinese Dietary Reference Intakes (2013) (DRIs-2013) (Zhang et al., 2012), all subjects were divided into two dietary nutrient consumption patterns which were labeled higher nutrient consumption pattern (the average intake of nutrients is higher than the reference) and lower nutrient consumption pattern (the average intake of nutrients is lower than the reference).

### Cognitive Function Assessment

A comprehensive neuropsychological battery test, on the basis of previous research, was adopted to assess the respondents’ cognitive performance. In this study, Montreal cognitive assessment-Beijing version (MoCA-BJ) was used to find out the MCI patients (Chen et al., 2016). Mini-mental state examination (MMSE) was used to evaluate the global cognitive decline (Piccinin et al., 2013). Short-term memory and working memory were assessed by digit span forward and digit span backward of the Wechsler adult intelligence test-revised Chinese version (Lu et al., 2017). Digit symbol subtest of WAIS-RC (Uchida et al., 2016) was used to assess executive function, attention and processing speed trail making test (Zhao et al., 2013) was made up of two parts. Part A was used to evaluate visual search and psychomotor speed. Meanwhile, Part B was used to assess set shifting, attention and executive function. The auditory verbal learning test (AVLT) (Zhao et al., 2015) was adopted to measure the capability of immediate recall, short recall and delayed recall. The investigation was conducted face to face by trained nurses and/or investigators in the local hospital. All cognitive function assessments were carried out according to the guidelines and protocols.

### Statistical Analysis

Statistical analyses were performed by SPSS version 24.0 for Windows (SPSS Inc., Chicago, IL, United States). All analyses were two-sided. The statistical significant level was *P* < 0.05. The data of continuous variables were shown in means ± standard deviation (SD) or medians (interquartile ranges, IQR). Discrete variables were expressed in percentage (%). One-way analysis of variance (ANOVA) or the Mann–Whitney *U*-test was used to detect the difference of variables. The frequencies variable was compared by Pearson χ^2^ or Fisher’s exact test. Dunnett *t*-tests and Bonferroni corrections were applied to the multiple comparisons. Logistic regression analysis (backward stepwise) and multiple linear regression analyses were used to examine the relationship between the different stages of early life Famine exposure and multi-dimensional cognitive function in late adulthood. The non-exposed group was the reference.

Model 1 was adjusted for age, sex, education, smoking, drinking, obesity, stroke, hypercholesterolemia, hypertension, HDL-C. Model 2 was further adjusted for the variables in Model 1 and dietary nutrient consumption patterns. The interaction between Famine exposure and dietary nutrient consumption patterns on cognitive decline was tested by adding a multiplicative factor in logistic regression model. Hierarchical analysis was adopted to explore whether the association of fetal and different childhood Famine exposure with cognitive function was modified by dietary nutrient consumption patterns in late adulthood.

## Results

### Sample Characteristics

The demographic characteristics of subjects in baseline survey were shown in **Table 1**. A total of 1162 participants were enrolled. 896 subjects, nearly 77%, had been exposed to the Chinese Famine during different life stages. Compared with the people in non-exposed group, the individuals in late childhood-exposed group had a lower education level, especially in terms of high school, undergraduate and higher grade education, their number keeps going down as the education level rises. The incidence of stroke and hypercholesterolemia of fetal exposed group was significantly higher than that of the non-exposed group. In addition, compared with the non-exposed group, the risks of obesity, hypercholesterolemia and over level of HDL-C were significantly higher in early childhood-exposed group. Meanwhile, the late childhood-exposed individuals also had higher risks of stroke and hypertension than non-exposed subjects.

**Table 1:** Demographic characteristic and clinical parameters of 1162 subjects.

	Non-exposed	Fetal-exposed	Childhood-exposed		
			
			Early childhood	Mid childhood	Late childhood
*N*	266	210	254	227	205
Birthdate^a^	1962–1964	1959–1961	1956–1958	1954–1956	1952–1954
Age in 2017	53–54	56–57	59–60	61–62	63–64
Gender, male, *n* (%)	126 (47.4)	83 (39.5)	124 (48.8)	109 (48.0)	93 (45.4)
Education, *n* (%)					
Illiterate	6 (2.3)	1 (0.5)	1 (0.4)	4 (1.8)	1 (0.5)
Primary school	29 (10.9)	15 (7.1)	29 (11.4)	26 (11.5)	52 (25.4)
Junior high school	102 (38.3)	65 (31.0)	99 (39.0)	99 (43.6)	87 (42.4)
High school	73 (27.4)	72 (34.3)	64 (25.2)	45 (19.8)	14 (6.8)
Junior college	37 (13.9)	40 (19.0)	43 (16.9)	37 (16.3)	39 (19.0)
Undergraduate and above	19 (7.1)	17 (8.1)	18 (7.1)	16 (7.0)	12 (5.9)
Smoking status, *n* (%)					
Never	186 (69.9)	158 (75.2)	182 (71.7)	160 (70.5)	146 (71.2)
Former	15 (5.6)	10 (4.8)	9 (3.5)	9 (4.0)	5 (2.4)
Current	65 (24.4)	42 (20.0)	63 (24.8)	58 (25.6)	54 (26.3)
Drinking status, *n* (%)					
Never	182 (68.4)	146 (69.5)	169 (66.5)	158 (69.6)	142 (69.3)
Former	14 (5.3)	17 (8.1)	20 (7.9)	14 (6.2)	18 (8.8)
Current	70 (26.3)	47 (22.4)	65 (25.6)	55 (24.2)	45 (22.0)
Family history, *n* (%)	26 (9.8)	20 (9.5)	20 (7.9)	17 (7.5)	13 (6.3)
Computer use, *n* (%)	111 (41.7)	72 (34.3)	81 (31.9)	80 (35.2)	62 (30.2)
Watching TV, *n* (%)	12 (4.5)	13 (6.2)	20 (7.9)	13 (5.7)	10 (4.9)
Reading, *n* (%)	82 (30.8)	65 (31.0)	80 (31.5)	75 (33.0)	74 (36.1)
Occupation, *n* (%)					
Manual	220 (82.7)	176 (83.8)	198 (78.0)	172 (75.8)	155 (75.6)
Non-manual	46 (17.3)	34 (16.2)	56 (22.0)	55 (24.2)	50 (24.4)
Labor intensity, *n* (%)					
Mild	180 (67.7)	150 (71.4)	178 (70.1)	171 (75.3)	159 (77.6)
Moderate	73 (27.4)	53 (25.2)	62 (24.4)	47 (20.7)	40 (19.5)
Strong	13 (4.9)	7 (3.3)	14 (5.5)	7 (3.3)	6 (2.9)
Work time, *n* (%)					
1	156 (58.6)	128 (61.0)	146 (57.5)	140 (61.7)	126 (61.5)
2	64 (24.1)	48 (22.9)	52 (20.5)	32 (14.1)	29 (14.1)
3	8 (3.0)	10 (4.8)	12 (4.7)	8 (3.5)	3 (1.5)
4	38 (14.3)	24 (11.4)	44 (17.3)	47 (20.7)	47 (22.9)
CHD, *n* (%)	18 (6.8)	19 (9.0)	13 (5.1)	20 (8.8)	20 (9.8)
Stroke, *n* (%)	1 (0.4)	10 (4.8)^∗^	2 (0.8)	3 (1.8)	9 (4.4)^∗^
Obesity, *n* (%)	20 (7.5)	24 (11.4)	40 (15.7)^∗^	30 (13.2)	20 (9.8)
Hypertension, *n* (%)	77 (28.9)	61 (29.0)	71 (28.0)	77 (33.9)	86 (42.0)^∗^
Abdominal obesity, *n* (%)	171 (64.3)	129 (61.4)	150 (59.1)	131 (57.7)	131 (63.9)
Hypercholesterolemia, *n* (%)	41 (15.4)	55 (26.2)^∗^	63 (24.8)^∗^	39 (17.2)	47 (22.9)
BMI	24.6 ± 2.9	24.6 ± 3.3	24.5 ± 2.9	24.7 ± 3.0	24.7 ± 3.6
TC	4.29 ± 1.01	4.28 ± 1.00	4.42 ± 1.08	4.29 ± 0.94	4.32 ± 0.99
TG	1.49 (1.12, 2,06)	1.50 (1.11, 2,13)	1.55 (1.09, 2.12)	1.58 (1.17, 2.18)	1.62 (1.20, 2.17)
HDL-C	1.20 (1.04, 1.40)	1.22 (1.06, 1.42)	1.30 (1.10, 1.48)^∗^	1.20 (1.08, 1.40)	1.26 (1.10, 1.43)
LDL-C	2.57 (2.03, 3.10)	2.49 (2.00, 3.11)	2.60 (2.10, 3.23)	2.58 (2.10, 3.05)	2.57 (2.01, 3.11)


*Work time (h/week): 1 <40; 2, 41–50; 3, 51–60; and 4, >60; CHD, coronary heart disease; BMI, body mass index; TC, total cholesterol; TG, triglyceride; HDL-C, high density lipoprotein cholesterol; LDL-C, low density lipoprotein cholesterol. ^a^From 1 October year to 30 September year. Continuous variables are shown as mean ± standard, analysis of variance or Mann–Whitney *U*-test were used for continuous variables with a normal distribution and a skewed distribution when comparing differences. Categorical variables are shown as *n* (%), using the Pearson χ^*2*^ test when comparing differences. ^∗^*P* < 0.0125 (Bonferroni correction) was considered to be statistically significant.*

### Early Stages of Life Exposed to the Chinese Famine and Cognitive Function in Adulthood

**Table 2** presents the subjects’ performances of cognitive tests in each group. Compared with non-exposed group, the fetal-exposed subjects had significantly lower score in the test of MoCA-BJ, immediately recall of AVLT and digit symbol. Early childhood-exposed individuals had a lower score of digital span backward test. In addition, mid childhood-exposed subjects had lower scores of immediately and short recall of AVLT, but the score of trail making test B was higher than non-exposed group. However, no significant differences have been found among the groups in the tests of MMSE, delayed recall of AVLT and digit span forward.

**Table 2:** Performance on cognitive tests in Famine exposure groups.

	Non-exposed	Fetal-exposed	Childhood-exposed		
			
			Early childhood	Mid childhood	Late childhood
MoCA-BJ	25 (23, 27)	24 (22, 26)^∗^	25 (22, 27)	25 (22, 26)	25 (23, 27)
MMSE	29 (27, 30)	28 (27, 29)	28 (27, 29)	28 (27, 29)	29 (27, 30)
Immediately recall of AVLT	16.1 ± 5.0	14.7 ± 5.8^∗^	15.2 ± 5.5	14.6 ± 4.9^∗^	14.9 ± 5.5
Short recall of AVLT	6 (4, 7)	5 (4, 7)	5 (4, 7)	5 (4, 7)^∗^	5 (4, 6)
Delayed recall of AVLT	5 (4, 7)	4 (4, 6)	4 (3, 6)	4 (4, 6)	4 (4, 7)
Digit span forward	8 (7, 9)	7 (7, 8)	8 (7, 8)	8 (7, 8)	8 (7, 8)
Digit span backward	4 (4, 5)	4 (3, 4)	4 (3, 4)^∗^	4 (4, 4)	4 (4, 4)
Trail making test A	62 (48, 79)	63 (50, 84)	65 (49.5, 83)	66 (52, 84)	63 (50.3, 79)
Trail making test B	144 (108, 183)	156 (128, 205.5)	155 (120, 201)	162 (125, 210)^∗^	147 (120, 192.5)
Digit symbol	35.7 ± 9.8	33.2 ± 9.4^∗^	34.9 ± 9.8	34.8 ± 9.4	35.9 ± 9.6


*Fewer scores indicate better cognitive performance in trail making test A and B whereas higher scores indicate better cognitive performance in other tests. Data are presented as means ± SD and median (interquartile range). Analysis of variance or Mann–Whitney *U*-test were used for continuous variables with a normal distribution and a skewed distribution when comparing differences. ^∗^*P* < 0.05 was considered to be statistically significant.*

The results of logistic regression analysis showed that the occurrence of MCI and global cognitive decline had significant association with the Famine exposure in the different stages of early life (**Table 3**). Compared with the individuals in non-exposed group, the risk of MCI in the subjects of fetal-exposed group and early childhood-exposed group were 1.43 (95% CI 0.97–2.11, *P* = 0.069) and 1.48 (95% CI 1.02–2.15, *P* = 0.039), respectively, when the data adjusted for demographic and clinical characteristics (Model 1). Meanwhile, the risk of global cognition decline were 1.64 (95% CI 1.00–2.68, *P* = 0.051) and 1.78 (95% CI 1.10–2.84, *P* = 0.018). Further adjusted for dietary nutrient consumption patterns (Model 2), the risk of MCI and global cognitive decline in the subjects of fetal-exposed group increased significantly which were 1.51 (95% CI 1.02–2.23, *P* = 0.039) and 1.68 (95% CI 1.02–2.77, *P* = 0.044).

**Table 3:** The risk of MCI and global cognition decline in Famine exposure groups.

	Non-exposed	Fetal-exposed	Childhood-exposed		
			
			Early childhood	Mid childhood	Late childhood
**MCI**					
*N*	87/179	89/121	106/148	91/136	74/131
OR (95% CI)^a^	Ref.	1.43 (0.97–2.11)	1.48 (1.02–2.15)	1.40 (0.96–2.06)	1.44 (0.95–2.17)
*p*^a^	Ref.	0.069	0.039^∗^	0.085	0.086
OR (95% CI)^b^	Ref.	1.51 (1.02–2.23)	1.53 (1.05–2.22)	1.45 (0.99–2.14)	1.49 (0.98–2.26)
*p*^b^	Ref.	0.039^∗^	0.026^∗^	0.058	0.061
**Global cognitive decline**					
*N*	36/230	45/165	54/200	40/187	24/181
OR (95% CI)^a^	Ref.	1.64 (1.00–2.68)	1.78 (1.10–2.84)	1.38 (0.84–2.82)	0.95 (0.54–1.70)
*p*^a^	Ref.	0.051	0.018^∗^	0.209	0.87
OR (95% CI)^b^	Ref.	1.68 (1.02–2.77)	1.80 (1.12–2.90)	1.40 (0.84–2.32)	0.94 (0.52–1.68)
*p*^b^	Ref.	0.044^∗^	0.016^∗^	0.193	0.82


*MCI, mid cognitive impairment; OR, odds ratio; Ref., reference group. All ORs use non-exposed group as reference group. ^a^Model 1 adjusted for age, sex, education, smoking, drinking, obesity, stroke, hypercholesterolemia, hypertension and HDL-C. ^b^Model 2 adjusted for the variables in model 1 and dietary nutrient intake combination patterns. ^∗^*P* < 0.05 was considered to be statistically significant.*

Multiple linear regression analysis was used to detect the association between the performance of multiple domains cognitive tests and the Famine exposure in different stages of early life (**Table 4**). After adjusted for demographic, clinical and dietary nutrient consumption patterns (Model 2), the MoCA-BJ score in fetal-exposed group, early childhood-exposed group and mid childhood-exposed group were decreased by 1.036, 0.719, and 0.754 than that in the non-exposed group. The score of MMSE in fetal-exposed group and early childhood-exposed group were decreased by 0.448 and 0.512, respectively. Interestingly, compared with the adjustment of Model 1, further adjusted for dietary nutrient consumption patterns (Model 2) could increase the risk of delayed recall of AVLT in the subjects of mid childhood-exposed group. The same adjusted result was also found in the risk of digital span forward in fetal-exposed group. Moreover, different stages of Famine exposure could lead to worse performance of immediate recall of AVLT, short recall of AVLT, digit span forward, digit span backward, trail making test B and digital symbol compared with the non-exposed group. However, there is no association between Famine exposure and trail making test A.

**Table 4:** Performance on overall and specific cognitive tests in Famine exposure groups.

	Non-exposed	Fetal-exposed	Childhood-exposed		
			
			Early childhood	Mid childhood	Late childhood
**MoCA-BJ**					
Median (IQR)	25 (23, 27)	24 (22, 26)	25 (22, 27)	25 (23, 26)	25 (23, 27)
B^a^	Ref.	-0.950	-0.685	-0.696	-0.446
*P*^a^	Ref.	0.002^∗^	0.017^∗^	0.018	0.142
B^b^	Ref.	-1.036	-0.719	-0.754	-0.504
*P*^b^	Ref.	0.001^∗^	0.012^∗^	0.011^∗^	0.100
**MMSE**					
Median (IQR)	29 (27, 30)	28 (27, 29)	28 (27, 29)	28.5 (27, 29)	29 (28, 30)
B^a^	Ref.	-0.431	-0.502	-0.246	0.056
*P*^a^	Ref.	0.020^∗^	0.004^∗^	0.172	0.764
B^b^	Ref.	-0.448	-0.512	-0.265	0.058
*P*^b^	Ref.	0.016^∗^	0.004^∗^	0.143	0.756
**Immediately recall of AVLT**					
Mean ± SD	16.1 ± 5.0	14.7 ± 5.8	15.2 ± 5.5	14.6 ± 4.9	14.9 ± 5.5
B^a^	Ref.	-1.352	-0.816	-1.453	-1.198
*P*^a^	Ref.	0.006^∗^	0.078	0.002^∗^	0.015^∗^
B^b^	Ref.	-1.332	-0.851	-1.529	-1.265
*P*^b^	Ref.	0.007^∗^	0.067	0.001^∗^	0.011^∗^
**Short recall of AVLT**					
Median (IQR)	6 (4, 7)	5 (4, 7)	5 (4, 7)	5 (4, 7)	5 (4, 7)
B^a^	Ref.	-0.430	-0.442	-0.600	-0.269
*P*^a^	Ref.	0.047^∗^	0.031^∗^	0.005^∗^	0.217
B^b^	Ref.	-0.434	-0.444	-0.635	-0.283
*P*^b^	Ref.	0.046^∗^	0.031^∗^	0.003^∗^	0.198
**Delayed recall of AVLT**					
Median (IQR)	4 (4, 6)	4 (4, 6)	4 (3, 6)	4 (3, 6)	4 (4, 7)
B^a^	Ref.	-0.229	-0.291	-0.405	-0.213
*P*^a^	Ref.	0.293	0.159	0.057	0.329
B^b^	Ref.	-0.226	-0.297	-0.434	-0.220
*P*^b^	Ref.	0.303	0.152	0.042^∗^	0.319
**Digital span forward**					
Median (IQR)	8 (7, 9)	7 (7, 8)	8 (7, 8)	8 (7, 8)	8 (7, 8)
B^a^	Ref.	-0.156	0.617	0.800	1.100
*P*^a^	Ref.	0.476	0.125	0.131	0.096
B^b^	Ref.	-0.226	-0.095	-0.148	-0.111
*P*^b^	Ref.	0.026^∗^	0.327	0.136	0.282
**Digital span backward**					
Median (IQR)	4 (4, 4)	4 (3, 4)	4 (3, 4)	4 (4, 4)	4 (4, 4)
B^a^	Ref.	-0.258	-0.226	-0.181	-0.096
*P*^a^	Ref.	0.003^∗^	0.007^∗^	0.034^∗^	0.279
B^b^	Ref.	-0.256	-0.236	-0.179	-0.089
*P*^b^	Ref.	0.004^∗^	0.005^∗^	0.038^∗^	0.318
**Trail making test A**					
Median (IQR)	61 (49, 80)	63 (49.5, 84)	65 (49.5, 83)	65 (52, 83.5)	63 (50, 79)
B^a^	Ref.	2.139	2.673	3.257	0.308
*P*^a^	Ref.	0.074	0.237	0.161	0.898
B^b^	Ref.	2.704	3.146	3.764	0.775
*P*^b^	Ref.	0.256	0.164	0.105	0.748
**Trail making test B**					
Median (IQR)	143 (108, 183)	157 (129, 207)	153 (119.5, 193.5)	159 (125, 210)	145 (120, 193)
B^a^	Ref.	11.917	14.334	18.185	3.292
*P*^a^	Ref.	0.046^∗^	0.002^∗^	0.002^∗^	0.585
B^b^	Ref.	13.841	15.930	19.960	4.472
*P*^b^	Ref.	0.020^∗^	0.005^∗^	0.001^∗^	0.458
**Digital symbol**					
Mean ± SD	35.7 ± 9.8	33.2 ± 9.4	34.9 ± 9.8	34.8 ± 9.4	35.9 ± 9.6
B^a^	Ref.	-2.485	-0.826	-0.939	-0.283
*P*^a^	Ref.	0.006^∗^	0.337	0.287	0.754
B^b^	Ref.	-2.733	-0.917	-1.029	0.267
*P*^b^	Ref.	0.003^∗^	0.286	0.242	0.768


*Data are presented as means ± SD and median (interquartile range). ^a^Model 1 adjusted for age, sex, education, smoking, drinking, obesity, stroke, hypercholesterolemia, hypertension and HDL-C. ^b^Model 2 adjusted for the variables in model 1 and dietary nutrient intake combination patterns. ^∗^*P* < 0.05 was considered to be statistically significant.*

### Early Stages of Life Exposed to the Chinese Famine and Dietary Nutrients Intake in Adulthood

Because the risk of worse performance in specific cognition was increased after further adjusted for dietary nutrient consumption patterns (**Tables 3**, **4**), the interaction analysis were used in present study to investigate the moderator role of dietary nutrient consumption pattern in the relationship between Famine exposure in early life and cognitive decline in adulthood. Data showed that there was significant interaction trend between dietary nutrient consumption pattern and Famine exposure on global cognition decline (*P* = 0.056, **Table 5**).

**Table 5:** The interaction of dietary nutrient consumption pattern and Famine exposure on the risk of MCI and global cognition decline.

	Non-exposed	Fetal-exposed	Childhood-exposed		
			
			Early childhood	Mid childhood	Late childhood
**MCI**					
OR(95% CI)	Ref.	1.51 (1.02–2.23)	1.53 (1.05–2.22)	1.45 (0.99–2.14)	1.49 (0.98–2.26)
*p*^a^	Ref.	0.039^∗^	0.026^∗^	0.058	0.061
*P* for interaction	Ref.	0.170	0.158	0.278	0.531
**Global cognitive decline**					
OR (95% CI)	Ref.	1.68 (1.02–2.77)	1.80 (1.12–2.90)	1.40 (0.84–2.32)	0.94 (0.52–1.68)
*p*^a^	Ref.	0.044^∗^	0.016^∗^	0.193	0.82
*P* for interaction	Ref.	0.964	0.967	0.056	0.894


*MCI, mid cognitive impairment; OR, odds ratio; Ref., reference group. All ORs use non-exposed group as reference group. ^a^Model 2 adjusted for age, sex, education, smoking, drinking, obesity, stroke, hypercholesterolemia, hypertension, HDL-C and dietary nutrient intake combination patterns. *P* for interaction: considerate the interaction between dietary nutrient consumption pattern and Famine exposure on MCI/Global cognitive decline based on adjusted for model 2. ^∗^*P* < 0.05 was considered to be statistically significant.*

After the interaction analysis, further nutrients exploration was carried out in this study. The analysis of nutrients consumption included protein, fat, carbohydrate, dietary fiber, cholesterol, vitamin A, carotene, retinol, thiamin, riboflavin, niacin, vitamin C, vitamin E, folic acid, calcium, phosphorus, potassium, sodium, magnesium, iron, zinc, selenium, copper, manganese, iodine, fatty acid, saturated fatty acid (SFA), monounsaturated fatty acids (MUFA), polyunsaturated fatty acids (PUFA). Compared with non-exposed group, the consumption of fat was much higher in the subjects of fetal-exposed group and early childhood-exposed group (*P* < 0.05). In addition, the individuals in late childhood-exposed group consumed more carbohydrate and manganese (*P* < 0.05) (**Table 6**). The other nutrients were not shown in this manuscript because there were no significant differences among groups. Meanwhile, there is no association between Famine exposure and dietary nutrient consumption patterns.

**Table 6:** The dietary nutrients intake in Famine exposure groups.

Nutrients	Non-exposed	Fetal-exposed	Childhood-exposed		
			
			Early childhood	Mid childhood	Late childhood
Fat (g)	40.7 ± 1.1	48.3 ± 1.6^∗^	48.3 ± 1.5^∗^	42.9 ± 1.5	46.6 ± 1.5
Carbohydrate (g)	209.0 ± 5.4	211.4 ± 6.1	203.1 ± 5.5	205.8 ± 5.7	229.6 ± 6.5^∗^
Manganese (mg)	7.7 ± 0.2	8.2 ± 0.3	7.8 ± 0.2	7.8 ± 0.3	8.7 ± 0.3^∗^
**Consumption pattern**					
1	103 (40.3)	87 (41.8)	97 (38.2)	83 (36.7)	94 (46.5)
2	157 (59.7)	121 (58.2)	157 (61.8)	143 (63.3)	108 (53.5)


*Consumption pattern: 1, high nutrient consumption pattern; 2, low nutrient consumption pattern. Continuous variables were presented as mean ± SE, using the analysis of variance when comparing differences. Categorical variables are shown as *n* (%), using the Pearson χ^*2*^ test when comparing differences. ^∗^*P* < 0.0125 (Bonferroni correction) was considered to be statistically significant.*

### The Correlation of Early Life Famine Exposure, Adult Dietary Nutrients Intake, and Cognitive Decline in Adulthood

As shown in **Table 7**, after adjusted for the demographic and clinical characteristics, the daily consumption of fat in adulthood could influence the scores of trail making test A (increase 0.362 points), trail making test B (increase 0.629 points) and digital symbol (decrease 0.084 points) in late childhood-exposed subjects. The daily intake of carbohydrate could decrease by 0.01 and 0.003 points in the scores of MoCA-BJ and digital span backward in fetal-exposed group. Meanwhile, the consumption of carbohydrate could decrease by 0.003 points in the score of digital span forward in early childhood-exposed group. The intake of manganese could increase by 0.032 points in the score of MoCA-BJ in fetal-exposed subjects and increase by 0.084 points in digital span forward in late childhood-exposed subjects. No significant differences were observed among the consumption of fat, carbohydrate, and manganese with the performance of MMSE, immediately recall of AVLT, short recall of AVLT, and delayed recall of AVLT in different Famine exposed groups.

**Table 7:** The correlation among Famine exposure, cognitive function and dietary nutrients intake.

	Group	Fat		Carbohydrate		Manganese	
				
		B	*P*	B	*P*	B	*P*
MoCA-BJ							
	Non-exposed	-0.018	0.238	-0.005	0.299	0.129	0.295
	Fetal-exposed	0.003	0.835	-0.010	0.017^∗^	0.223	0.032^∗^
	Early childhood	-0.008	0.463	-0.005	0.243	0.099	0.329
	Mid childhood	0.003	0.816	-0.008	0.082	0.048	0.675
	Late childhood	-0.024	0.086	0.009	0.069	-0.067	0.551
MMSE							
	Non-exposed	-0.004	0.637	0.002	0.463	-0.050	0.505
	Fetal-exposed	-0.006	0.502	-0.003	0.343	0.079	0.281
	Early childhood	-0.005	0.427	-0.002	0.473	0.046	0.465
	Mid childhood	-0.002	0.822	0.003	0.347	-0.004	0.948
	Late childhood	0.007	0.410	0.005	0.091	-0.080	0.204
Immediately recall of AVLT							
	Non-exposed	0.004	0.859	-0.009	0.224	0.224	0.244
	Fetal-exposed	0.004	0.850	-0.006	0.468	-0.005	0.977
	Early childhood	-0.014	0.387	0.000	0.985	-0.114	0.466
	Mid childhood	-0.007	0.732	-0.004	0.586	0.057	0.729
	Late childhood						
Short recall of AVLT							
	Non-exposed	-0.010	0.280	-0.002	0.507	0.050	0.520
	Fetal-exposed	-0.010	0.280	-0.002	0.507	0.050	0.520
	Early childhood	-0.003	0.650	0.004	0.181	-0.113	0.111
	Mid childhood	0.004	0.687	-8.450	0.979	0.039	0.610
	Late childhood	0.002	0.863	0.004	0.229	-0.083	0.315
Delayed recall of AVLT							
	Non-exposed	-0.005	0.629	-0.004	0.313	0.166	0.071
	Fetal-exposed	-0.006	0.491	-0.001	0.743	0.043	0.571
	Early childhood	-0.003	0.655	0.004	0.248	-0.110	0.126
	Mid childhood	0.005	0.550	-0.002	0.534	0.038	0.623
	Late childhood	-0.002	0.808	0.006	0.101	-0.081	0.313
Digital span forward							
	Non-exposed	-0.004	0.462	0.002	0.245	-0.031	0.474
	Fetal-exposed	0.003	0.505	-0.001	0.397	0.042	0.249
	Early childhood	0.003	0.363	-0.003	0.041^∗^	0.053	0.072
	Mid childhood	-1.523	0.997	0.001	0.670	0.001	0.987
	Late childhood	0.006	0.212	-0.005	0.006^∗^	0.084	0.045^∗^
Digital span backward							
	Non-exposed	0.007	0.109	0.000	0.929	-0.010	0.768
	Fetal-exposed	0.006	0.126	-0.003	0.037^∗^	0.056	0.089
	Early childhood	0.001	0.847	0.000	0.857	0.003	0.919
	Mid childhood	0.000	0.962	0.001	0.370	-0.032	0.299
	Late childhood	-0.005	0.213	0.001	0.517	-0.001	0.964
Trail making test A							
	Non-exposed	0.119	0.344	0.010	0.795	-0.443	0.665
	Fetal-exposed	0.001	0.989	0.021	0.546	-0.380	0.650
	Early childhood	0.101	0.195	0.046	0.169	-0.918	0.221
	Mid childhood	-0.008	0.939	0.018	0.615	0.464	0.589
	Late childhood	0.362	0.001^∗^	-0.039	0.316	-0.516	0.562
Trail making test B							
	Non-exposed	0.201	0.505	0.053	0.580	-1.759	0.474
	Fetal-exposed	0.408	0.097	0.036	0.659	-1.640	0.408
	Early childhood	0.237	0.241	0.119	0.169	-1.280	0.512
	Mid childhood	0.099	0.710	0.123	0.214	-1.151	0.623
	Late childhood	0.629	0.019^∗^	-0.019	0.836	-1.968	0.351
Digital symbol							
	Non-exposed	-0.006	0.908	-0.017	0.269	0.305	0.433
	Fetal-exposed	0.009	0.807	-0.021	0.103	0.297	0.336
	Early childhood	-0.023	0.439	-0.007	0.568	0.170	0.564
	Mid childhood	0.001	0.983	0.013	0.348	-0.413	0.208
	Late childhood	-0.084	0.048^∗^	0.025	0.096	-0.292	0.389


*Data are presented as means ± SD and median (interquartile range). Adjusted for age, sex, education, smoking, drinking, obesity, stroke, hypercholesterolemia, hypertension and HDL-C. ^∗^*P* < 0.05 was considered to be statistically significant.*

**FIGURE 1:**
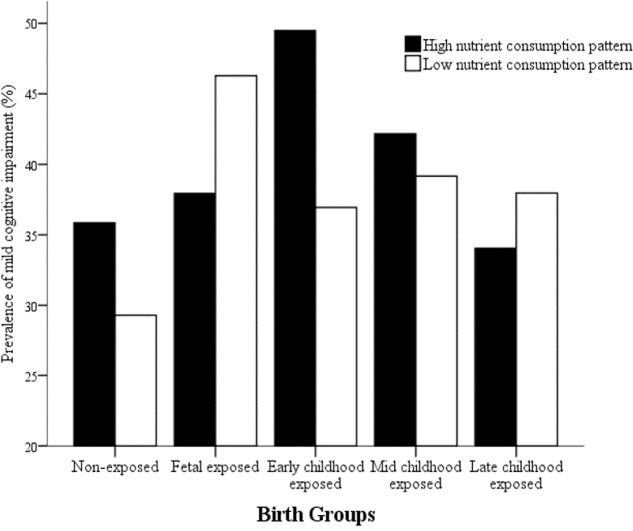
The effects of exposure to Chinese Famine during different life stages of early life on the prevalence of mild cognitive impairment (MCI) stratified by dietary nutrient consumption patterns. The prevalence of MCI was detected by the Montreal cognitive assessment-Beijing version (MoCA-BJ).

**FIGURE 2:**
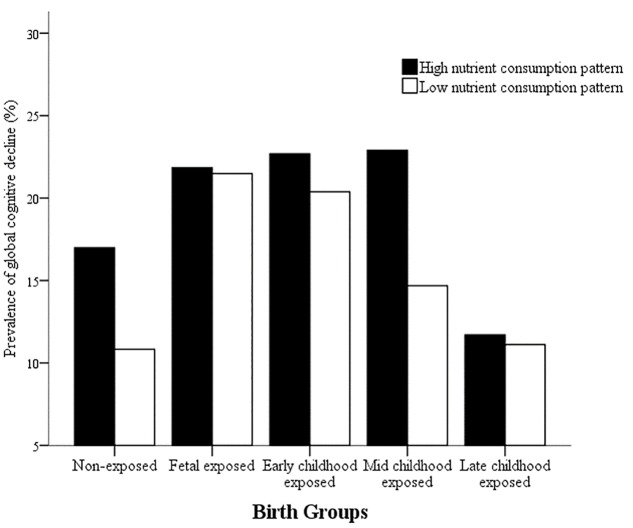
The effects of exposure to Chinese Famine during different life stages of early life on the prevalence of global cognitive decline stratified by dietary nutrient consumption patterns. The prevalence of global cognitive decline was detected by the mini-mental state examination (MMSE).

In order to detect whether dietary nutrient consumption pattern is an important factor affecting the correlation between early life Famine exposure and cognitive decline in adulthood, hierarchical analysis was used in this study (**Figures 1**, **2**). The subjects in early childhood-exposed group with higher nutrient consumption pattern had a particularly high prevalence of MCI (49.5%), whereas the prevalence in the subjects with lower nutrient consumption pattern was only 36.9% (**Figure 1**). As shown in **Figure 2**, the prevalence of global cognitive decline had a higher trend in the subjects with higher nutrient consumption pattern in fetal-exposed group (21.8%), early childhood-exposed group (22.7%), mid childhood-exposed group (22.9%) and late childhood-exposed group (10.8%) than the people with lower nutrient consumption pattern. But there is no significant difference. **Table 8** showed the performance of cognitive tests between two dietary nutrient consumption patterns. The scores of trail making test B were manifestly different in two patterns (*P* < 0.05).

**Table 8:** Performance on cognitive tests in different dietary nutrient consumption patterns.

	Pattern 1	Pattern 2	*t*	*p*
MoCA-BJ	24.0 ± 3.4	24.4 ± 3.2	-1.792	0.073
MMSE	27.9 ± 2.1	28.0 ± 2.0	-1.192	0.233
Immediately recall of AVLT	14.9 ± 5.3	15.3 ± 5.3	-1.431	0.153
Short recall of AVLT	5.5 ± 2.3	5.5 ± 2.4	0.043	0.966
Delayed recall of AVLT	4.9 ± 2.3	5.0 ± 2.4	-0.59	0.556
Digital span forward	7.8 ± 1.1	7.6 ± 1.1	1.947	0.052
Digital span backward	4.1 ± 1.0	4.0 ± 0.9	1.033	0.302
Trail making test A	70.2 ± 26.1	67.4 ± 25.1	1.798	0.072
Trail making test B	171.5 ± 69.9	163.4 ± 60.3	2.031^∗^	0.043
Digital symbol	34.6 ± 9.7	35.2 ± 9.6	-1.067	0.286


*Pattern 1: high nutrient consumption pattern; Pattern 2, low nutrient consumption pattern. Data are presented as means ± SD using the Student’s *t*-test when comparing differences. ^∗^*P* < 0.05 was considered to be statistically significant.*

## Discussion

It is demonstrated by ‘developmental origins of adult health and disease hypothesis’ (Kent, 2012; Charles, 2013) that experienced malnutrition, hormone exposure or other unfavorable factors in early stages of life may lead to procedural and permanent changes in the structure and function of tissues and organs. These changes may result in increased risk of various chronic disease and neurodegenerative diseases in adulthood. Therefore, it is emphasized that nutrition in early stage of life is a vital factor for human health in later life. In recent years, epidemiological studies and animal experiments have suggested that early life malnutrition may lead to cognitive decline in adulthood (de Rooij et al., 2010; Reynolds, 2013). However, the specific mechanisms of this relation are not yet clear. Meanwhile, the opposite opinions make the association still in controversy. Thus, further evidences need to be explored by researchers. Data of our previous study has shown that there is an association of long-term health consequences with prenatal and postnatal life exposed to the Chinese Famine (Wang C. et al., 2016). In this study, the results indicated that malnutrition in different stages of early life has a profound and long-term influence on overall and specific cognitive decline in adulthood. The Chinese Famine exposure could lead to increased risk of cognitive decline in late life. Moreover, the association is stronger in the subjects who had higher nutrient consumption pattern.

The fetal origin hypothesis indicates that prenatal and postnatal malnutrition could increase the risk of neurodegenerative disease in late life. Researchers demonstrated that the brain size was determined by the conditions of fetal phase and first 2 years after birth. Malnutrition during the very beginning of life could result in a permanent deficit to brain structures. Animal study showed that cell proliferation and brain-derived neurotrophic factor level in hypothalamic and hippocampal could be affected in pregnant Wistar rats with 50% food restriction (FR50) which is believed having the association with the reduction of learning and memory ability (Coupé et al., 2009). In population research, Famine provides researchers a natural opportunity to verify this relationship. Studies on the long-term health effects of Dutch famine exposure demonstrated that prenatal malnutrition could negatively influence the cognitive capability in late adulthood (Rooij et al., 2010; Crookston et al., 2011). The Amsterdam study found that people who exposed to Dutch famine in prenatal period had worse selective attention performance tested by Stroop-like task (de Rooij et al., 2010). Similar to the results of Dutch famine, data in present study advocated that fetal and different childhood Famine exposure could influence the adult cognitive function. Since cognitive function is not a single clinical manifestation, it always appears in multiple domains. The results of this study also supported that different stages of early life malnutrition not only affect the overall cognitive decline, but also lead to the impairment of specific cognitive decline including working memory, long-term memory, executive function, attention, and processing speed which were tested by digital span backward, AVLT, digital symbol and trail making test. However, different opinion in the study of Dutch famine has shown that there is no association between Famine exposure and cognitive decline (de Groot et al., 2011). The reason of this divergence might be related to the age of the population, the proportion of sex, education level, test tools, etc.

What attributes to the correlation between early life malnutrition and adult cognitive decline? Some researches indicated that malnutrition during the very beginning of life not only had an irreversible impact on catch-up growth, but also had the susceptibility of chronic non-communicable disease in middle and later age (Lucas, 2010). The Dutch famine birth cohort study and Leningrad Siege Study suggested pregnancy Famine exposure was associated with higher risk of diabetes, obesity and depression in adulthood (Brown and Susser, 2008; Rooij, 2013; Hajj et al., 2014). As expected, the results in current study also supported that malnutrition in early life could increase the risk of stroke, hypertension, obesity, and hypercholesterolemia in our subjects. These chronic non-communicable diseases are recognized as the important risk factors for cognitive decline. Evidence showed that cognitive function in obese patients was particularly impaired in the domains of executive function and working memory (Stillman et al., 2017). It is known that adult dietary nutrition is a very important influencing factor of chronic non-communicable disease in later life. Therefore, we further propose another question: what is the role of adult dietary nutrition in the correlation between early life malnutrition and adult cognitive decline?

To test adult dietary nutrition as the influencing factor in the relation of early life malnutrition and adult cognitive decline, we first did the adjustment of dietary nutrient consumption pattern in the comparison of groups. The results of **Tables 3**, **4** suggested that the nutrient consumption patterns might be an important influencing factor in the relation between Famine exposure in early life and cognitive decline in adulthood. Then, we further did the interaction analysis between Famine exposure and dietary nutrient consumption pattern on cognitive decline. The *P*-value of interaction was very close to 0.05, it might be associated with the fewer subjects in this study. Therefore, the result was accepted as there is significant interaction trend on global cognition decline. Based on this potential moderator role of dietary nutrient consumption patterns, we did the further comparison of nutrients intake between groups. Evidence from Dutch famine birth cohort presented early gestation exposed to Famine were associated with a more atherogenic lipid profile and dietary preference of fatty foods (OR 2.1 95% CI 1.2–3.9) in male and female (aged 58 years), although no significant correlation was found in the study (Lussana et al., 2007). The Dutch famine study also suggested that the prevalence of obesity was higher in the first one-half of gestation exposed to Famine, and it was associated with elevation of HDL cholesterol and total cholesterol (Lumey et al., 2009). In current study, data showed that subjects exposed to Famine in early childhood had a higher risk of obesity in adulthood. Consistent with this result, present study also found that people who exposed to Famine in early childhood and in fetal period had a higher daily intake of fat in adulthood. This might be the reason of higher risk of obesity in the individuals of early childhood-exposed group. In addition, the person who exposed to Famine in later childhood had the tendency of higher intake of fat in adulthood, but no significant difference was found in this study. Analyzing the correlation of fat intake and cognitive function, the result showed that subjects with a higher consumption of fat had a higher risk of worse performance on trail making test A, trail making test B and digital symbol in late childhood-exposed group. Similar to the demonstration of Dutch famine birth cohort study (Uchida et al., 2016), our results implied that people who experienced early life malnutrition might be prone to high-fat diet especially in the people of later childhood malnutrition exposure, and the habit of higher intake of fatty food might be a vital factor in the association between early life malnutrition and adult cognitive decline. Besides no significant correlation was found between fat intake and cognitive decline in early childhood-exposed group. This might be associated with the small sample size of present study.

Moreover, micronutrients such as zinc, iron, copper, and aluminum have been demonstrated having association with cognitive function, but the results were controversial (Mclachlan et al., 1996; Martyn et al., 1997; Modgil et al., 2014). Numerous people-based study suggested iron, zinc, iodine, folates and B vitamins might improve cognitive performance (Nyaradi et al., 2015; Shah-Kulkarni et al., 2016; Portbury et al., 2017). In contrary, zinc and copper were believed to result in β-amyloid peptides aggregation which is one of the major pathological markers in the brain of AD patients (ElAli et al., 2013; Crăciun et al., 2016). The reason of this inconsistent might be inappropriate analysis methods, different selection of sample and matching controls (Modgil et al., 2014). In present study, it was found that the consumption of manganese in late childhood-exposed subjects were higher than non-exposed individual. Besides the micronutrients, the daily intake of carbohydrate in the people of late childhood-exposed group was higher than in the non-exposed group. These two nutrients were linked with MoCA-BJ, digital span forward and digital span backward in different groups. The results implied that adult dietary nutrients intake could influence the cognitive decline in the people suffering early life malnutrition, especially in the late childhood exposure subjects. Our results were consistent with the fetal programming ‘mismatch’ hypothesis, which is late life environment could modify the association of early life malnutrition with the health consequences.

The study of ‘mismatch’ hypothesis indicated that when the people exposed to Famine in early life and facing ‘rich’ environment later, the risk of chronic disease maybe increase (Li et al., 2011a). There was evidence that association between BMI and blood pressure was stronger among individuals who were exposed to the Famine during fetal life in severely affected area and had a western dietary pattern in later life (Li et al., 2011a). In addition, it was demonstrated that higher risk of metabolic syndrome was in the people who suffered Chinese Famine in fetal period and had a western dietary pattern in later life (Li et al., 2011b). In this study, the subjects in early childhood-exposed group with higher nutrient consumption pattern had a particularly high prevalence of MCI. The prevalence of global cognitive decline had a higher trend in the subjects with higher nutrient consumption pattern in Famine exposed groups than in non-exposed group. Moreover, the people with higher nutrient consumption patterns had worse performance on set shifting, attention and executive function. These results implied that the adult nutritional ‘rich’ environment did not match the early life malnutrition environment, which might increase the risk of cognitive decline in adulthood.

## Conclusion

In summary, present study, as a result of our phased study, indicated that different stages of early life exposed to Famine was associated with higher risk of cognitive decline in adulthood. The people suffering early life malnutrition might be prone to high-fat diet, especially in the people of later childhood malnutrition exposure, and the habit of higher intake of fatty food might be a vital factor in the association between early life malnutrition and adult cognitive decline. This conclusion needs more evidence with large scale of samples, and it will be the topic we focus on in the future study of our team. The nutritional ‘rich’ environment in adulthood which was represented by a higher dietary nutrient consumption pattern could increase the susceptibility of cognitive decline in late life. The consumption of fat, carbohydrate and manganese was related to the cognitive function in adulthood. The current study suggested that both early life and adult nutritional environments were critical for the cognitive function in late life.

## Author Contributions

RX and ZQ designed the study. XZ, HY, and YW carried out all the experiments. HR, YA, and LT contributed to analysis and interpretation of data. HR and YX analyzed the experimental results and wrote the manuscript. All authors critically reviewed the final version of the manuscript.

## Conflict of Interest Statement

The authors declare that the research was conducted in the absence of any commercial or financial relationships that could be construed as a potential conflict of interest.
